# Not All Brugada Electrocardiogram Patterns are Brugada Syndrome or Brugada Phenocopy

**DOI:** 10.4274/balkanmedj.2017.0621

**Published:** 2017-12-01

**Authors:** Grace Xu, Byron H. Gottschalk, Umut Kocabaş, Adrian Baranchuk

**Affiliations:** 1 Department of Medicine, Queen’s University School of Medicine, Kingston, Canada; 1 Department of Cardiac Sugery, Western University School of Medicine, Ontario, Canada; 1 Clinic of Cardiology, Soma State Hospital, Manisa, Turkey

We have recently read the case report by Arı and Ekici (1) with great interest and noted that this patient displayed electrocardiogram patterns characteristic of Brugada syndrome after a propafenone overdose with the intention of suicide. This case report is an important contribution to the investigation of the aetiology of Brugada electrocardiogram patterns and their classification as Brugada syndrome, Brugada phenocopies, or other diagnoses. We have published extensively on the topic of Brugada phenocopies (2,3) and would like to clarify the terminology used in classifying the case report to avoid confusion in future research.

Antiarrhythmic agents block sodium channels and may be used to unmask true Brugada syndrome at appropriate dosages (4). The class 1C antiarrhythmic agent, propafenone, taken at equivalent doses, may serve a similar role to other sodium channel blockers but has not been systematically tested. Although the effects of large doses have not been systematically investigated, we suspect that overdoses of propafenone may induce artificial sodium channel dysfunction even without genetic predisposition, causing a Brugada electrocardiogram pattern.

The term Brugada phenocopies does not cover abnormal electrocardiogram patterns that are induced by sodium channel blockers (2). Thus, overdose using propafenone would not be considered Brugada phenocopies and instead, may be classified as an “acquired sodium channel dysfunction”.

We thank Dr. Arı and Ekici for using the term Brugada phenocopies in their literature; however, we would like to emphasise that Brugada electrocardiogram patterns induced by sodium channel blockers should not be classified as Brugada phenocopies. We are continuing the development of our international online registry at www.brugadaphenocopy.com and encourage investigators working on Brugada phenocopies to submit their cases online to provide long-term follow up and investigation of the natural history of this condition.
